# Polyphenols Isolated from *Xanthoceras sorbifolia* Husks and Their Anti-Tumor and Radical-Scavenging Activities

**DOI:** 10.3390/molecules21121694

**Published:** 2016-12-09

**Authors:** Chun-Yan Yang, Wei Ha, Yong Lin, Kan Jiang, Jun-Li Yang, Yan-Ping Shi

**Affiliations:** 1 CAS Key Laboratory of Chemistry of Northwestern Plant Resources and Key Laboratory for Natural Medicine of Gansu Province, Lanzhou Institute of Chemical Physics, Chinese Academy of Sciences, Lanzhou 730000, China; yangaccn@163.com (C.-Y.Y.); hawei2012@licp.cas.cn (W.H.); jiangk15@licp.cas.cn (K.J.); 2 School of Pharmacy, Institute of Materia Medica, North Sichuan Medical College, Nanchong 637007, China; 3 Department of Preventive Medicine, North Sichuan Medical College, Nanchong 637007, China; lin-yong@vip.sina.com

**Keywords:** *Xanthoceras sorbifolia*, polyphenols, extracts, anti-tumor activity, radical-scavenging activity

## Abstract

*Xanthoceras sorbifolia* Bunge. is used in traditional medicine in North China. To evaluate the anti-tumor and radical-scavenging activities of *X. sorbifolia* husks polyphenols and determine their structure-activity relationships, 37 polyphenols **1**–**37** were obtained by bioassay-guided fractionation. Two new compounds **1**–**2**, and compounds **5**, **6**, **8**, **9**, **11**, **14**–**17**, **21**–**25**, **27**–**29**, **31**, **33**, **34**, **36**, and **37** were isolated from the genus *Xanthoceras* for the first time. Compounds **1**–**37** did not show strong cytotoxicity against the four tested tumor cell lines (A549, HepG2, MGC-803, and MFC) compared to paclitaxel and under the conditions tested in the anti-tumor assay, but compounds **3**, **4**, **7**, **8**, **10**, **18**–**20**, **25**, **26**, **29**, **30**, **32**, and **35** exhibited stronger radical-scavenging activity than ascorbic acid in a 2,2′-azino-bis(3-ethylbenzothiazoline-6-sulfonic acid) diammonium salt assay. This was the first report on the anti-tumor and radical-scavenging activities of the polyphenols isolated from *X. sorbifolia* husks. Overall, the present study contributed valuable information concerning *X. sorbifolia* husks use in medicine and pharmacology.

## 1. Introduction

*Xanthoceras sorbifolia* Bunge. is the only species in the genus *Xanthoceras* (family Sapindaceae) and is distributed in North China [1]. This species is a kind of woody oil-bearing crop, traditionally used in herbal medicine for curing atherosclerosis, rheumatism, hyperpiesia, chronic hepatitis, and child enuresis [2], and it has been included in the 1977 edition of the China Pharmacopoeia [3]. Chemical studies of *X. sorbifolia* husks, which are considered byproducts, showed that the husks contained a variety of compounds, including polyphenols [4], triterpenoids [5], and sterols [6]. Medical research showed that *X. sorbifolia* husk components improved learning ability and memory [7], had anti-cancer effects [8], inhibited tyrosinase [9], cured cardiovascular diseases [10], had anti-oxidant properties [11], and inhibited pancreatic lipase activity [12]. However, anti-tumor and radical-scavenging activities have not yet been reported for the polyphenols isolated from *X. sorbifolia* husks.

Cancer is a multi-step disease that often involves the activation of oncogenes or the inactivation of tumor-suppressor genes. In addition to genetic mutations, different free radicals interfering with enzyme structure or activity are also responsible for cancer development [13]. In particular, growth factors, such as the platelet-derived growth factor and the epidermal growth factor, which can be activated by cancer cells to sustain cellular growth and proliferation, could rapidly, and transiently, increase reactive oxygen species (ROS) generation through nicotinamide adenine dinucleotide phosphate oxidases [14]. ROS are often associated with oxidative stress, which has been related to the progression of many diseases, including cancer and cardiovascular diseases (e.g., atherosclerosis) [15]. Thus, it is necessary to develop and utilize natural radical-scavenging and antitumor agent with low cytotoxicity so that they can help human get rid of over-produced ROS and also reduce the risks of suffering from cancer. Polyphenols, including flavonoids and phenolic acids, have been associated with protection against oxidative stress and cancer risk reduction [16]. In the present study, 37 polyphenols were isolated from *X. sorbifolia* husks, and their anti-tumor and radical-scavenging capacities were analyzed, together with those of the husks’ 70% aqueous ethanol extract, chloroform fraction, *n*-butanol fraction, and water fraction. The SAR of the polyphenols were also discussed.

## 2. Results and Discussion

### 2.1. Structure Elucidation

**Figure 1:**
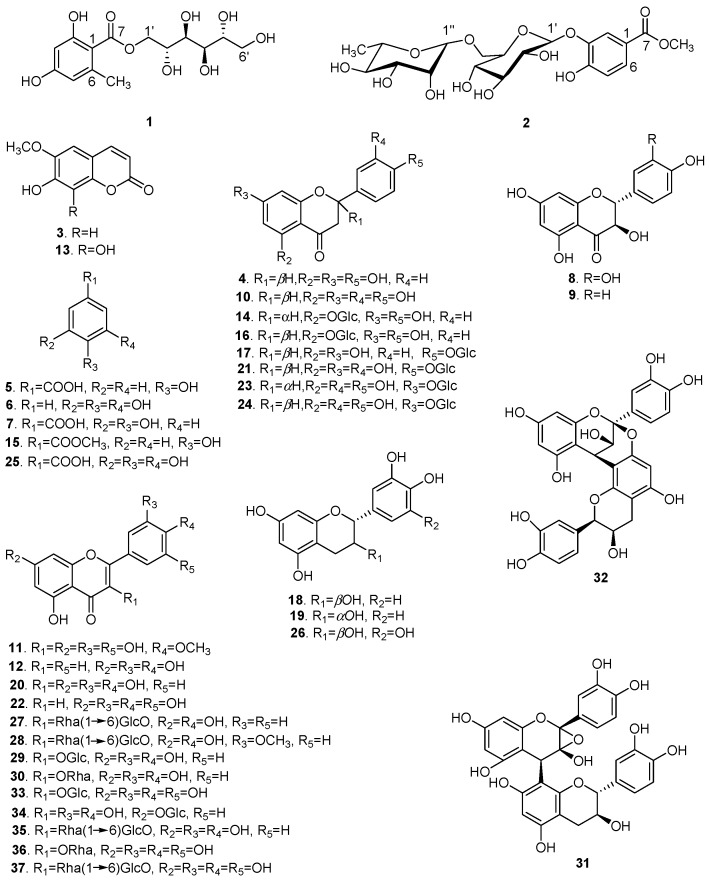
The chemical structures of polyphenols isolated from *X. sorbifolia* husks.

Repeated column chromatography using silica gel, Sephadex LH-20, and preparative high performance liquid chromatography (prep-HPLC) of the 70% aqueous ethanol extract of XS husks resulted in the isolation of 37 polyphenols with purities over 98% (Figure 1). Compound **1** was obtained as a yellowish gum ([α]${}_{D}^{20}$ +2.041°; *c* 0.49, MeOH). Its UV spectrum revealed absorption at *λ*_max_ 289 and 263 nm and the infrared (IR) spectrum suggested the presence of hydroxyl (3356.46 cm^−1^), alkyl (2919.23 cm^−1^), and olefin (1617.76 cm^−1^) groups. The molecular formula was determined as C_14_H_20_O_9_ by high resolution electrospray ionization mass spectrometry (HR-ESI-MS) at *m/z* 355.1003 [M + Na]^+^ (calcd. for C_14_H_20_O_9_Na, 355.1000), with five degrees of unsaturation. In the proton nuclear magnetic resonance (^1^H-NMR; Table 1), one tetrasubstituted phenyl [δ_H_ 6.18 (1H, br s) and 6.15 (1H, br s)] and one methyl (δ_H_ 2.37 (3H, s)) were easily recognized.

**Figure 2:**
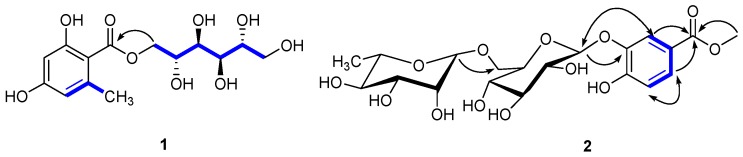
Key HMBC (H⟶C), ^1^H-^1^H COSY (−), and NOESY (**⟷**) of new compounds **1**, **2** isolated from *X. sorbifolia* husks.

The carbon nuclear magnetic resonance (^13^C-NMR) spectrum also showed six carbon signals at δc 67.7 (C-1′), 68.1 (C-2′), 69.7 (C-3′), 69.3 (C-4′), 71.2 (C-5′), and 63.8 (C-6′), suggesting the presence of a d-mannitol moiety [17]. The hetero-nuclear single quantum coherence (HSQC) spectroscopy identified one carbonyl (δc 170.2), one phenyl (δc 162.1, 161.5, 141.9, 110.6, 106.5, and 100.5), two oxygenated methylenes (δc 67.7, 63.8), and four oxygenated methines (δc 71.2, 69.7, 69.3, and 68.1). These data suggested that **1** was an orsellinic acid analog. The hetero-nuclear multiple bond correlation (HMBC) confirmed the presence of an orsellinic acid scaffold: H-3/C-1 (δc 106.5), C-2 (δc 162.1), C-5 (δc 110.6), H-5/C-4 (δc 161.5), C-1 (δc 106.5), C-3 (δc 100.5), CH_3_-6/C-6 (δc 141.9), C-5 (δc 110.6), C-1 (δc 106.5), and C-3 (δc 100.5) (Figure 2). The nuclear Overhauser effect spectroscopy (NOESY) correlation between CH_3_-6/H-5 and the ^1^H-NMR [δ_H_ 6.18 (1H, br s) and 6.15 (1H, br s)] results were in agreement with the substitution patterns of orsellinic acid. The location of the mannitol moiety at C-7 was deduced from the H-1′/C-7 HMBC correlation. Accordingly, the structure of **1** was determined as d-mannitol orsellinate, trivially named xspolyphenol A.

Compound **2** was obtained as a yellowish gum ([α]${}_{D}^{20}$ −52.055°; *c* 0.73, MeOH). The UV spectrum suggested absorption at λ_max_ 320 nm and the IR spectrum suggested the presence of hydroxyl (3356.07 cm^−1^), methyl (2918.26 cm^−1^), carbonyl (1676.87 cm^−1^), and aryl (1617.71, 1578.08 cm^−1^) groups. The molecular formula of **2** was determined as C_20_H_28_O_13_ by HR-ESI-MS at *m/z* 499.1418 [M + Na]^+^ (calcd, 499.1422), suggesting seven degrees of unsaturation. The ^1^H-NMR (Table 2) of compound **2** showed one ABX coupling system at: δ_H_ 7.39 (d, *J* = 2.8 Hz), 6.94 (d, *J* = 9.2 Hz) and 7.25 (dd, *J* = 2.8, 8.8 Hz) for H-2, H-5, and H-6 of a benzene ring, respectively, indicating the presence of a C-1,3,4 trisubstituted benzene moiety. In the ^1^H-NMR, the sharp signal at δ_H_ 3.90 (3H, s) suggested a –OCH_3_. In the HMBC spectrum (Figure 2), –OCH_3_ was assigned at C-7, due to the long-range coupling of C-7 and –OCH_3_. After the acidic hydrolysis of compound **2**, the aqueous layer was separated by thin-layer chromatography (TLC) to yield two glycosides: rhamnose and glucose. The position of the sugar linkage was assigned at C-3 by HMBC correlations (Figure 2) and confirmed by the positive NOESY between H-2 (δ_H_ 7.39) and H-1′ (δ_H_ 4.68). The configurations of the anomeric protons of **2** were deduced to be α and β forms based on the ^3^*J*_H1,H2_ coupling conditions (H-1′′ (brs) and H-1′′′′′ (*J* = 7.6 Hz)). Accordingly, the chemical structure of **2** was unambiguously established as methyl 4-hydroxylbenzoate 3-*O*-*α*-l-rhamnopyranosyl-(1→6)-β-d-glucopyranoside, trivially named xspolyphenol B.

Based on spectroscopic data and by comparison to previously reported compounds, Compounds **3**–**37** were identified as: scopoletin (**3**) [18], naringenin (**4**) [19], *p*-hydroxybenzoic acid (**5**) [20], pyrogallol (**6**) [21], protocatechuic acid (**7**) [22], taxifolin (**8**) [23], aromadendrin (**9**) [24], eriodictyol (**10**) [25], mearnsetin (**11**) [26], luteolin (**12**) [27], fraxetin (**13**) [28], naringenin 5-*O*-β-d-glucopyranoside (**14**) [29], methyl 4-hydroxylbenzoate (**15**) [30], (−)-salipurposide (**16**) [31], naringenin 4′-*O*-β-d-glucopyranoside (**17**) [32], (+)-catechin (**18**) [33], epicatechin (**19**) [34], quercetin (**20**) [34], eriodictyol 4′-*O*-β-d-glucopyranoside (**21**) [35], tricetin (**22**) [36], (2*S*)-eriodictyol 7-*O*-β-d-glucopyranoside (**23**) [37], (2*R*)-eriodictyol 7-*O*-β-glucopyranoside (**24**) [38], gallic acid (**25**) [39], gallocatechin (**26**) [40], kaempferol 3-*O*-rutinoside (**27**) [41], isorhamnetin 3-*O*-rutinoside (**28**) [42], isoquercitrin (**29**) [43], quercitrin (**30**) [44], 2α,3α-epoxy-5,7,3′,4′-tetrahydroxyflavan-(4β-8-catechin) (**31**) [45], proanthocyanidin A2 (**32**) [46], isomericitrin (**33**) [47], quercimetrin (**34**) [48], rutin (**35**) [34], myricetrin (**36**) [44], and myricetin 3-*O*-rutinoside (**37**) [49].

### 2.2. Chemotaxonomic Significance

The polyphenols identified from *X. sorbifolia* husks provided an image concerning the chemotaxonomic situation of the genus *Xanthoceras* within the family Sapindaceae. The main polyphenols isolated from *X. sorbifolia* husks were protocatechuic acid (6.01 mg/100 g husks), epicatechin (5.24 mg/100 g husks), catechin (3.34 mg/100 g husks), rutin (2.81 mg/100 g husks), myricetin-3-*O*-rutinoside (1.37 mg/100 g husks), quercetin (1.19 mg/100 g husks), and quercitrin (1.12 mg/100 g husks); quercetin and myricetin were the major aglycons in *X. sorbifolia* husks. Previous phytochemical studies of the genus showed that their polyphenolic pool comprised mostly of flavonoids and phenolic acids [4], but these compounds were not quantified. The present study is the first to isolate and quantify the polyphenolic compounds from *X. sorbifolia* husks, which might be important for the chemotaxonomy of the genus and family.

### 2.3. Anti-Tumor Effects of X. sorbifolia Polyphenols

**Figure 3:**
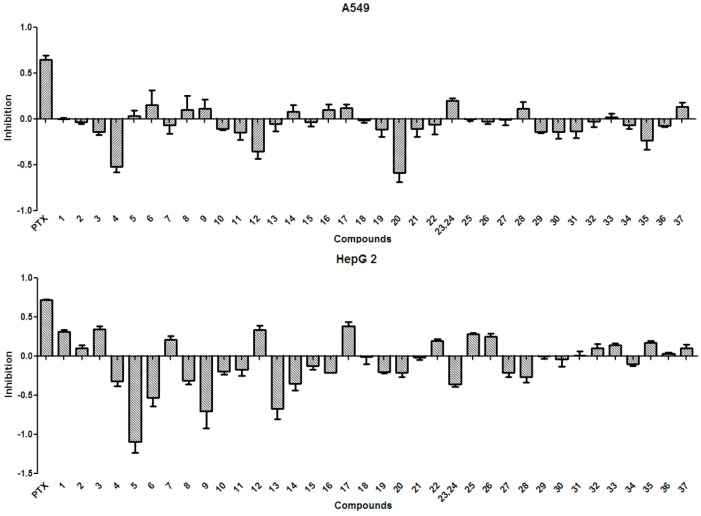
The anti-tumor activity against A549 and HepG2 of the polyphenols (**1**–**37**) isolated from *X. sorbifolia* husks. MTT 3-(4,5-dimethyl-2-thiazolyl)-2,5-diphenyltetrazolium bromide was dissolved in PBS at 5 mg/mL. After the formazans were dissolved in DMSO for 10 min, the absorbance values at 490 nm were measured, and the percentage inhibitions were calculated. PTX, Paclitaxel.

**Figure 4:**
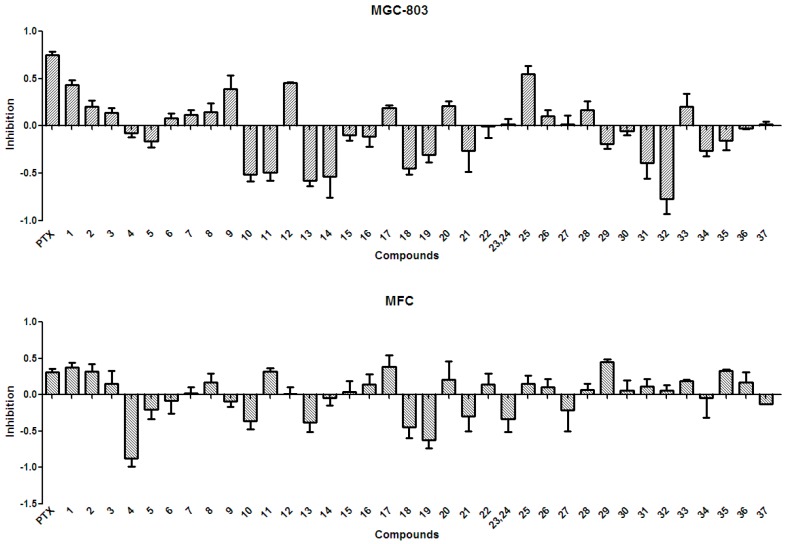
The anti-tumor activity against MGC-803 and MFC of the polyphenols (**1**–**37**) isolated from *X. sorbifolia* husks. MTT 3-(4,5-dimethyl-2-thiazolyl)-2,5-diphenyltetrazolium bromide was dissolved in PBS at 5 mg/mL. After the formazans were dissolved in DMSO for 10 min, the absorbance values at 490 nm were measured, and the percentage inhibitions were calculated. PTX, Paclitaxel.

In general, the anti-tumor activities of natural products are evaluated by testing their ability to directly inhibit the proliferation of tumor cells, or their capacity to induce immune cells to secrete cytokines that could act on tumor cells [15]. Tests for the anti-tumor activity of the polyphenols isolated from the husks (**1**–**37**) at a concentration of 50 µg/mL, revealed that all compounds had null or weak cytotoxicity, when compared to that of paclitaxel and under the conditions described in the present study. The percentage inhibition of compounds **1**–**37** at 50 µg/mL against four tumor cell lines is summarized in Figure 3 and Figure 4. Protocatechuic acid (**7**) showed no effect on the lung adenocarcinoma (A549) cell line, similarly to that described in a previous study [50]; however, in that study, protocatechuic acid (**7**) exhibited anti-tumor activity against other cancer cell lines, at high concentrations. Previous studies [34,51] also reported that epicatechin (**19**) had no effect or a weak effect on the A549, liver cancer (HepG2), and gastric carcinoma (MGC-803) cell lines, supporting the results found in the present study. It has also been previously reported [52] that catechin (**18**) had a weak effect on HepG2 and MGC-803 cell lines. Figure 3 and Figure 4 evidence that rutin (**35**) exhibited no or weak effects on A549, HepG2, and MGC-803 cell lines, in agreement with previous reports [13,34,53]. Although myricetin 3-*O*-rutinoside (**37**) showed no or weak anti-tumor activity against A549, HepG2, MGC-803, and murine foregastric carcinoma (MFC) cell lines (as shown in Figure 3 and Figure 4), it was firstly investigated in the present study. Similar to that found in a previous study [33], quercetin (**20**) had no effect on the MGC-803 cell line. As there were few reports on the in vitro anti-tumor activity of polyphenols on the MFC cell line, the present study represents a major addition to the knowledge on this subject. Overall, polyphenols isolated from *X. sorbifolia* husks did not strongly inhibit the proliferation of some cancer cell lines, compared to paclitaxel, under the conditions examined in this research. Interestingly, we found that almost half of the compounds exhibited negative inhibition effects, the mechanism was worthy of further study and could be studied by other tumor cell lines or even animal models.

### 2.4. Radical-Scavenging Activity of Extracts and Polyphenols

#### 2.4.1. Radical-Scavenging Activity

**Figure 5:**
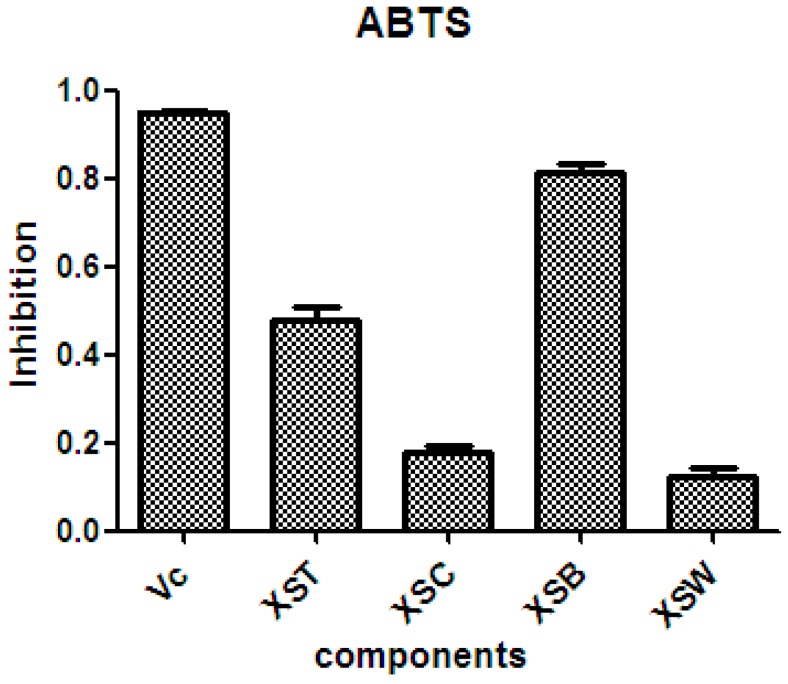
The radical-scavenging activity of the crude samples from *X. sorbifolia* husks. ABTS was prepared daily and diluted to an absorbance of 0.70 ± 0.02 at 734 nm. After the crude samples (50 µg/mL) reacted with the ABTS radical solution for 10 min, the absorbance values (*Ai*) at 734 nm were measured, and the percentage inhibitions were calculated. Vc, ascorbic acid; XST, 70% aqueous ethanol extract; XSC, chloroform soluble fraction; XSB, *n*-butanol soluble fraction; XSW, water soluble fraction. PTX, Paclitaxel.

**Figure 6:**
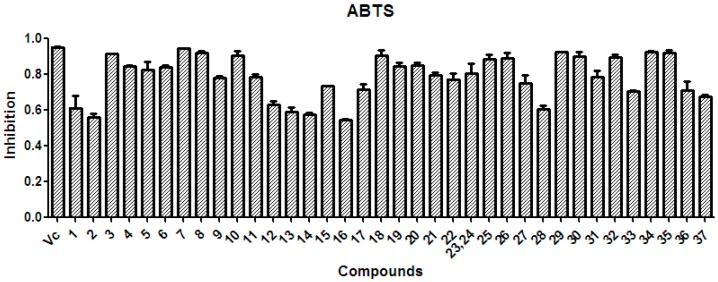
The radical-scavenging activity of the polyphenols (**1**–**37**) isolated from *X. sorbifolia* husks. ABTS was prepared daily and diluted to an absorbance of 0.70 ± 0.02 at 734 nm. After the crude samples (50 µg/mL) reacted with the ABTS radical solution for 10 min, the absorbance values (*Ai*) at 734 nm were measured, and the percentage inhibitions were calculated. Vc, ascorbic acid.

There is considerable evidence that free radicals induce oxidative damage to biomolecules and play an important role in cancer and cardiovascular diseases [54]. The 2,2′-azino-bis(3-ethyl-benzothiazoline-6-sulfonic acid) diammonium salt (ABTS) assay has been a popular radical-scavenging test for natural components [55] and, therefore, it was used in the present study. The XSB fraction presented the highest radical-scavenging activity among *X. sorbifolia* husk extracts (Figure 5). Then the bioassay-guided fractionation of *X. sorbifolia* husks led to the isolation of 37 polyphenols. The evaluation on the radical-scavenging activity of the polyphenols at a concentration of 50 µg/mL showed that compounds **1**–**37** had strong radical-scavenging activity (Figure 6), and, therefore, all compounds were subject to ABTS assay to determine their scavenging capability. As shown in Figure 6 and Table 3, the main polyphenols (protocatechuic acid, epicatechin, (+)-catechin, rutin, myricetin-3-*O*-rutinoside, quercetin, and quercitrin) exhibited strong radical-scavenging activities, compared to ascorbic acid. Thus, the husks’ main compounds strongly contributed to the striking radical-scavenging activity of the XSB fraction. As free radical damage is indicated to be the main cause of cancer [56], although *X. sorbifolia* polyphenols did not strongly inhibited the proliferation of cancer cell lines under the conditions described in the present research (Figure 3 and Figure 4), they might indirectly present anti-tumor activity by reducing oxidative damage [57].

#### 2.4.2. Discussion on the SAR of Polyphenols

The polyphenols isolated from *X. sorbifolia* husks can be divided into three categories (Figure 1): flavonoids, phenolic acids, and coumarins. The twenty-eight flavonoids can be further classified into flavonols, flavones, flavanones, flavan-3-ols, and flavanonols. Flavonoids with vicinal phenolic hydroxyls presented strong radical-scavenging activity, in agreement with that previously reported [58]. In comparison with the flavonoids bearing saccharide groups, different characteristics of the sugar side chain also play important roles in their radical–scavenging effect. The flavonoids **36**, **37** possess the same aglycone, but flavonoid glycoside **37** with a disaccharide chain exhibited weaker radical–scavenging effect than **36** with a monosaccharide chain, which suggested that the presence of a disaccharide chain might reduce the radical–scavenging effect. Thus, the antioxidant activities of these flavonoids would depend on not only the substituent groups on the aglycone, but also the sugar moieties. Phenolic acids with both carboxyl and vicinal phenolic hydroxyls also showed strong radical-scavenging activity. However, coumarins with a single phenolic hydroxyl exhibited stronger radical-scavenging activity than coumarins with vicinal phenolic hydroxyls.

## 3. Materials and Methods

### 3.1. General Experimental Procedures

Column chromatography was conducted using silica gel (SiO_2_, 200–300 µm mesh; Qingdao Haiyang Chemical Co., Ltd., Qingdao, China) and Sephadex LH-20 (20–100 µm; Pharmacia, Uppsala, Sweden) as packing materials. Silica GF_254_ (10–40 mm) for TLC was supplied by the Qingdao Marine Chemical Factory, Qingdao, China. All TLC spots were visualized under UV light (254 nm) and stained with a 10% H_2_SO_4_ solution in ethanol, followed by heating. Optical rotations were recorded with a 341 polarimeter (Perkin-Elmer, Waltham, MA, USA) in a 1 dm cell. The UV spectra were measured on a UV-260 spectrophotometer (Shimadzu, Nishinokyo Kuwabara-cho, Nakagyo-ku, Kyoto, Japan) and the IR spectra were obtained on a NEXUS 670 FT-IR spectrometer (Nicolet, Madison, WI, USA). Nuclear magnetic resonance spectra were recorded on INOVA-400 (Varian, Palo Alto, CA, USA) and AVANCE III-400 (Bruker, Billerica, MA, USA) spectrometers. Chemical shifts were given on a δ (ppm) scale using tetramethylsilane as the internal standard. High-resolution electrospray ionization (ESI) mass spectrometry (MS) was carried out on a APEX II mass spectrometer (Bruker Daltonics, Billerica, MA, USA) and ESI-MS spectra were determined on a Bruker Daltonics Esquire 6000 spectrometer. The ABTS and 3-(4,5-dimethyl-2-thiazolyl)-2,5-diphenyltetrazolium bromide (MTT) used were obtained from Aladdin Industrial Co. (Shanghai, China).

### 3.2. Plant Material and Reagents

*X. sorbifolia* husks (Sapindaceae) used in the present study were collected in Gansu Province, China, in 2013, and authenticated by Associate Prof. Huanyang Qi (Lanzhou Institute of Chemical Physics, Chinese Academy of Sciences, Lanzhou, China). A voucher specimen (No. 20131106XSB) was deposited in the Key Laboratory of Chemistry of Northwestern Plant Resources and Key Laboratory for Natural Medicine of Gansu Province, Chinese Academy of Sciences. All chemicals were of industrial or analytical grade and used after redistillation.

### 3.3. Extraction and Isolation

After air-drying, *X. sorbifolia* husks (10.0 kg) were pulverized and refluxed with 70% aqueous ethanol at 65 °C (four times, using 100 L, for 2 h). Extracts were filtered and then concentrated under reduced pressure to yield the ethanol extract (XST; 2.0 kg). Most XST (1.95 kg) was then suspended in distilled water (18 L) and successively partitioned with chloroform (four times, using 6 L), and water-saturated *n*-butanol (four times, using 6 L) to yield the chloroform soluble fraction (XSC; 86.0 g), the *n*-butanol soluble fraction (XSB; 560.0 g), and the aqueous fraction (XSW; 1.3 kg). As XSB exhibited a marked radical-scavenging activity (Figure 6), this active fraction was subjected to sequential silica-gel chromatography, Sephadex LH-20, and prep-HPLC to obtain compounds **1**–**37** (see Supplementary Materials). Most XSB (500.0 g) was loaded into an ordinary-phase silica gel column (5 kg, 15 cm × 35 cm) with a CHCl_3_:MeOH elution gradient of 10:1, 5:1, 2:1, 1:1, and 1:1 (water saturated). The eluate was collected in 108 portions of 3000 mL, and eluates containing similar components according to the TLC results were combined into 11 fractions (Fr. 1 to Fr. 11).

Fr. 1 was chromatographed on a Sephadex LH-20 column eluted with CHCl_3_:MeOH (1:1) to obtain compounds **3** (10.0 mg) and **4** (8.0 mg).

Fr. 2 was chromatographed on a Sephadex LH-20 column eluted with CHCl_3_:MeOH (1:1), and four subfractions I–IV were obtained. Subfraction II was purified by prep-HPLC eluted with MeOH:H_2_O (42:58) to obtain compound **5** (t_R_ 10.0 min, 8.0 mg). Subfraction III was firstly purified by prep-HPLC eluted with MeOH:H_2_O (34:66) to obtain compounds **6** (t_R_ 7.3 min, 4.2 mg) and **7** (t_R_ 8.5 min, 601.3 mg), and then eluted with MeOH:H_2_O (43:57) to obtain compounds **8** (t_R_ 12.7 min, 8.4 mg), **9** (t_R_ 20.3 min, 10.0 mg), and **10** (t_R_ 31.2 min, 83.0 mg). Subfraction IV was purified by prep-HPLC eluted with MeOH:H_2_O (45:55) to obtain compounds **11** (t_R_ 55.0 min, 2.6 mg) and **12** (t_R_ 66.7 min, 3.4 mg).

Fr. 3 was chromatographed on a Sephadex LH-20 column eluted with CHCl_3_:MeOH (1:1), and five sub fractions I–V were obtained. Subfraction III was first purified by prep-HPLC eluted with MeOH:H_2_O (30:70) to obtain compounds **13** (t_R_ 20.8 min, 15.3 mg), **14** (t_R_ 34.2 min, 9.3 mg), **15** (t_R_ 38.4 min, 5.2 mg), and **16** (t_R_ 41.1 min, 9.2 mg); and then eluted with MeOH:H_2_O (36:64) to obtain compound **17** (t_R_ 39.9 min, 19.8 mg). Subfraction IV was purified by prep-HPLC eluted with MeOH:H_2_O (13:87) to obtain compounds **18** (t_R_ 21.6 min, 334.4 mg) and **19** (t_R_ 49.3 min, 523.8 mg). Subfraction V was purified by prep-HPLC eluted with MeOH:H_2_O (42:58) to obtain compound **20** (t_R_ 74.8 min, 118.5 mg).

Fr. 4 was chromatographed on a Sephadex LH-20 column eluted with CHCl_3_:MeOH (1:1), and four sub fractions I–IV were obtained. Subfraction III was purified by prep-HPLC eluted with MeOH:H_2_O (38:62) to obtain compound **21** (t_R_ 28.0 min, 10.0 mg) and subfraction IV was purified by prep-HPLC eluted with MeOH: H_2_O (43:57) to obtain compound **22** (t_R_ 60.8 min, 2.3 mg).

Fr. 5 was chromatographed on a Sephadex LH-20 column eluted with CHCl_3_:MeOH (1:1), and five sub fractions I–V were obtained. Subfraction III was first purified by prep-HPLC eluted with MeOH:H_2_O (33:67) to obtain compound **1** (t_R_ 13.5 min, 4.9 mg), and then eluted with MeOH:H_2_O (40:60) to obtain the isomers of **23** and **24** at a ratio of approximately 1:1 (t_R_ 18.0 min, 36.3 mg). Sub- fraction V was purified by prep-HPLC eluted with MeOH:H_2_O (10:90) to obtain compounds **25** (t_R_ 23.0 min, 38.7 mg) and **26** (t_R_ 37.7 min, 8.6 mg).

Fr. 6 was chromatographed on a Sephadex LH-20 column eluted with CHCl_3_:MeOH (1:1), and six subfractions I–VI were obtained. Subfraction III was first purified by prep-HPLC eluted with MeOH:H_2_O (30:70) to obtain compound **2** (t_R_ 27.0 min, 7.3 mg), and then eluted with MeOH:H_2_O (39:61) to obtain compounds **27** (t_R_ 64.9 min, 38.5 mg) and **28** (t_R_ 71.6 min, 17.4 mg). Subfraction IV was purified by prep-HPLC eluted with MeOH:H_2_O (35:65) to give compounds **29** (t_R_ 48.4 min, 35.8 mg) and **30** (t_R_ 70.2 min, 119.9 mg).

Fr. 7 was chromatographed on a Sephadex LH-20 column eluted with CHCl_3_:MeOH (1:1), and four subfractions I–IV were obtained. Subfraction III was purified by prep-HPLC eluted with MeOH:H_2_O (20:80) to obtain compound **31** (t_R_ 45.4 min, 4.0 mg) whereas subfraction IV was purified by prep-HPLC eluted with MeOH:H_2_O (30:70) to obtain compound **32** (t_R_ 66.0 min, 5.0 mg).

Fr. 8 was chromatographed on a Sephadex LH-20 column eluted with CHCl_3_:MeOH (1:1), and four sub fractions I–IV were obtained. Subfraction III was purified by prep-HPLC eluted with MeOH:H_2_O (38.5:61.5) to obtain compounds **33** (t_R_ 31.6 min, 14.5 mg) and **34** (t_R_ 36.4 min, 4.1 mg).

Fr. 9 was chromatographed on a Sephadex LH-20 column eluted with CHCl_3_:MeOH (1:1), and five subfractions I–V were obtained. Subfraction III was purified by prep-HPLC eluted with MeOH:H_2_O (40:60) to obtain compound **35** (t_R_ 29.0 min, 280.5 mg).

Fr. 10 was chromatographed on a Sephadex LH-20 column eluted with CHCl_3_:MeOH (1:1), and six subfractions I–VI were obtained. Subfraction III was purified by prep-HPLC eluted with MeOH:H_2_O (34.3:65.7) to obtain compound **36** (t_R_ 53.7 min, 7.8 mg).

Fr. 11 was chromatographed on a Sephadex LH-20 column eluted with CHCl_3_:MeOH (1:1), and six subfractions I–VI were obtained. Subfraction IV was purified by prep-HPLC eluted with MeOH:H_2_O (24:76) to give compound **37** (t_R_ 54.7 min, 137.1 mg).

### 3.4. Acidic Hydrolysis of the New Compound and Sugar Analysis

The new compound **2** (2 mg) was added to a solution of concentrated HCl (0.5 mL), and refluxed for 3 h, by adding H_2_O (1.5 mL)/dioxane (3 mL). After dilution with H_2_O, the reaction mixture was subjected to extraction twice with ethyl acetate (EtOAc). The H_2_O layers of compound **2** were then neutralized with NaHCO_3_ and concentrated to dryness under reduced pressure. The residue was re-dissolved in H_2_O for TLC analysis.

### 3.5. Biological Activity

#### 3.5.1. Anti-Tumor Assay

Compounds **1**–**37** were evaluated for their anti-tumor activity against three human cancer cell lines, the lung adenocarcinoma (A549), liver cancer (HepG2), and gastric carcinoma (MGC-803) cell lines, and one murine foregastric carcinoma (MFC) cell line, which were obtained from Shanghai Cell Bank of Chinese Academy of Sciences (Shanghai, China). Cells were cultured in Dulbecco’s modified eagle medium supplemented with 10% fetal bovine serum (both from Hyclone, South Logan, UT, USA), in 5% CO_2_ at 37 °C. The anti-tumor assay was performed according to the MTT method [58], with some modifications. In brief, A549, HepG2, MGC-803, and MFC cells were seeded into 96-well plates (5 × 10^3^ cells/well) for 20–24 h under the above conditions, treated with 50 µg/mL of each tested compound, and further incubated for 24 h under the same conditions. After this period, 20 µL of MTT stock solution (5 mg/mL in PBS) were added to each well and samples were incubated for another 4 h, under the same conditions. Supernatants were then removed, and 150 µL of dimethyl sulfoxide were added to each well. After 10 min, absorbance was determined on a Multiskan MK 3 Automated Microplate Reader (Thermo Fisher Scientific, Waltham, MA, USA) at 490 nm, using paclitaxel as the positive control. The percentage inhibition was calculated according to:

(1) AA(%)=[1−AiAo]×100%

where *AA* is the inhibition percentage, *Ao* is the absorbance of the blank sample, and *Ai* is the absorbance of the test sample.

#### 3.5.2. ABTS Radical-Scavenging Assay

Samples (extracts and polyphenols from *X. sorbifolia* husks) ability to scavenge the ABTS radical cation was measured following a previously reported method [59], with some modifications, and using L-ascorbic acid as the positive control. Assays were performed in 96-well plates and absorbance was measured at 734 nm. The radical-scavenging activity of each sample was expressed as the percentage inhibition of the ABTS radical and determined according to Equation (1).

### 3.6. Statistical Analysis

All experiments were carried out in three replicates to ensure reproducibility. Sample concentrations providing 50% scavenging capability (SC_50_) were obtained by fitting dose-response data to a four-parametric logistic nonlinear regression model, using GraphPad Prism 5.0 software (GraphPad, La Jolla, CA, USA).

## 4. Conclusions

In conclusion, quercetin and myricetin polyphenols were the main aglycons in *X. sorbifolia* husks, and this might be of great chemotaxonomic importance within the genus *Xanthoceras* and the family Sapindaceae. Pharmacological studies showed that, although compounds **1**–**37** did not show strong cytotoxicity against the four tumor cell lines (A549, HepG2, MGC-803, and MFC), compared to paclitaxel and under the conditions described in the present research, compounds **3**, **4**, **7**, **8**, **10**, **18**–**20**, **25**, **26**, **29**, **30**, **32**, and **35** showed stronger radical-scavenging activity than ascorbic acid in the ABTS assay. This was the first report on the anti-tumor and radical-scavenging activities of the polyphenols isolated from *X. sorbifolia* husks. The results obtained in the present study contribute important baseline information on the biological activity of *X. sorbifolia* husks, which might contribute for their pharmacological application.

## Figures and Tables

**Table 1:** ^1^H- and ^13^C-NMR data of the new compound **1** (in DMSO-*d*_6_, 400 MHz).

No.	^13^C-NMR	DEPT	^1^H-NMR
1	106.5	C	
2	162.1	C	
3	100.5	CH	6.15 (br s)
4	161.5	C	
5	110.6	CH	6.18 (br s)
6	141.9	C	
6-CH_3_	22.8	CH_3_	2.37 (s)
7	170.2	C	
1′	67.7	CH_2_	4.49 (dd, 1.6, 10.8), 4.24 (dd, 6.4, 11.2)
2′	68.1	CH	3.79 (m)
3′	69.7	CH	3.63 (m)
4′	69.3	CH	3.58 (m)
5′	71.2	CH	3.47 (m)
6′	63.8	CH_2_	3.62 (m), 3.40 (o)

Assignments were done by HSQC, HMBC, ^1^H-^1^H COSY and NOESY experiments; *J* values (Hz) are in parentheses; o: overlapped.

**Table 2:** ^1^H- and ^13^C-NMR data of the new compound **2** (in DMSO-*d*_6_, 400 MHz).

No.	^13^C-NMR	DEPT	^1^H-NMR
1	112.7	C	
2	117.0	CH	7.39 (d, 2.8)
3	155.4	C	
4	149.7	C	
5	118.2	CH	6.94 (d, 9.2)
6	125.5	CH	7.25 (dd, 2.8, 8.8)
7	168.9	C	
–OCH_3_	52.6	CH_3_	3.90 (s)
1′	101.9	CH	4.68 (d, 7.6)
2′	73.2	CH	3.20 (o)
3′	75.5	CH	3.42 (o)
4′	70.0	CH	3.08 (t, 8.8)
5′	76.3	CH	3.24 (m)
6′	66.6	CH_2_	3.85 (d, 9.2), 3.39 (o)
1′′	100.6	CH	4.54 (s)
2′′	70.3	CH	3.59 (br s)
3′′	70.7	CH	3.43 (o)
4′′	72.0	CH	3.16 (o)
5′′	68.3	CH	3.41 (o)
6′′	17.8	CH_3_	1.10 (d, 6.0)

Assignments were done by HSQC, HMBC, ^1^H-^1^H COSY and NOESY experiments; *J* values (Hz) are in parentheses; o: overlapped.

**Table 3:** Radical-scavenging activity of the compounds (**1**–**37**) isolated from *X. sorbifolia* husks.

Compounds	SC_50_^a^ Values (µg/mL)	Compound	SC_50_^a^ Values (µg/mL)
	ABTS		ABTS
**1**	>50	**20**	3.45 ± 1.38
**2**	12.59 ± 1.33	**21**	37.95 ± 1.87
**3**	4.72 ± 1.13	**22**	10.19 ± 1.11
**4**	5.99 ± 1.09	**23, 24**	12.73 ± 1.02
**5**	20.61 ± 1.07	**25**	3.65 ± 1.02
**6**	13.48 ± 1.08	**26**	4.76 ± 1.10
**7**	4.32 ± 1.09	**27**	32.31 ± 4.87
**8**	6.82 ± 1.18	**28**	28.65 ± 1.07
**9**	>50	**29**	5.69 ± 1.13
**10**	5.85 ± 1.04	**30**	5.25 ± 1.07
**11**	10.19 ± 1.11	**31**	8.48 ± 1.15
**12**	11.16 ± 1.05	**32**	5.23 ± 1.18
**13**	24.44 ± 1.16	**33**	13.86 ± 1.10
**14**	52.33 ± 1.15	**34**	7.99 ± 1.11
**15**	>50	**35**	7.46 ± 1.09
**16**	42.43 ± 1.54	**36**	9.05 ± 1.07
**17**	18.15 ± 1.27	**37**	14.04 ± 1.09
**18**	4.45 ± 1.06	Ascorbic acid ^b^	7.67 ± 1.09
**19**	4.54 ± 1.36		

^a^ The SC_50_ value of each compound was defined as the concentration (µg/mL) that caused 50% scavenging capability of ABTS; ^b^ Ascorbic acid was used as positive control.

## Supplementary Materials

Supplementary materials can be accessed at: http://www.mdpi.com/1420-3049/21/12/1694/s1.
