# Supplementary material for: Polyphenols Isolated from Xanthoceras sorbifolia Husks and Their Anti-Tumor and Radical-Scavenging Activities
Source: Molecules. 2016 Dec 9;21(12):1694. doi: 10.3390/molecules21121694 (PMC6274062; doi:10.3390/molecules21121694)
Supplement: Supplementary file 1 [file molecules-21-01694-s001.pdf]

Supplementary Materials: Polyphenols Isolated from *Xanthoceras sorbifolia* Husks and Their Anti-Tumor and Radical-Scavenging Activities

Chun-Yan Yang, Wei Ha, Yong Lin, Kan Jiang, Jun-Li Yang and Yan-Ping Shi

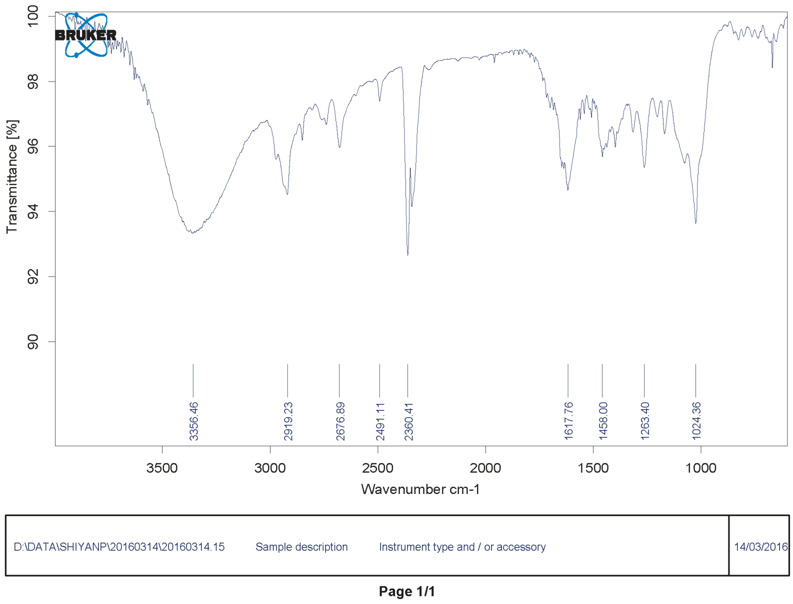

Figure S1. IR spectrum of xspolyphenol A.

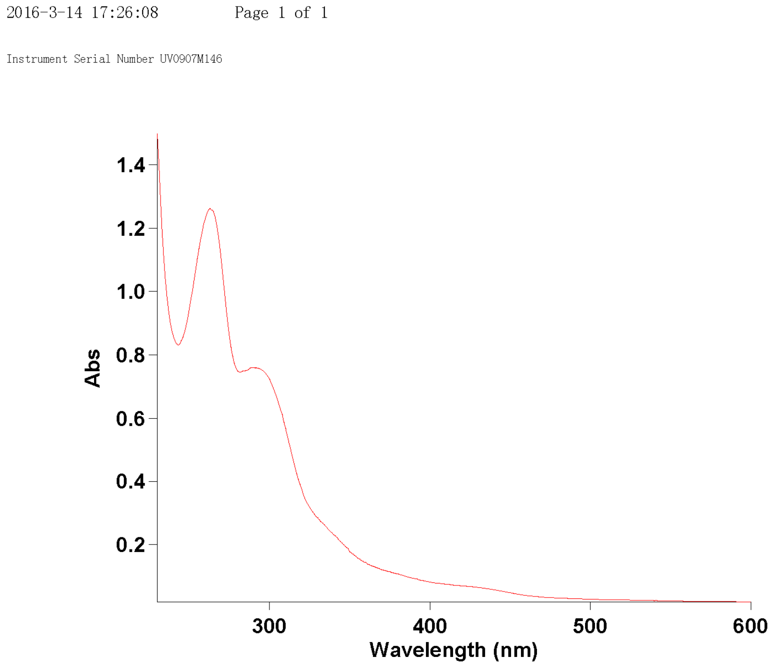

Figure S2. UV spectrum of xspolyphenol A.

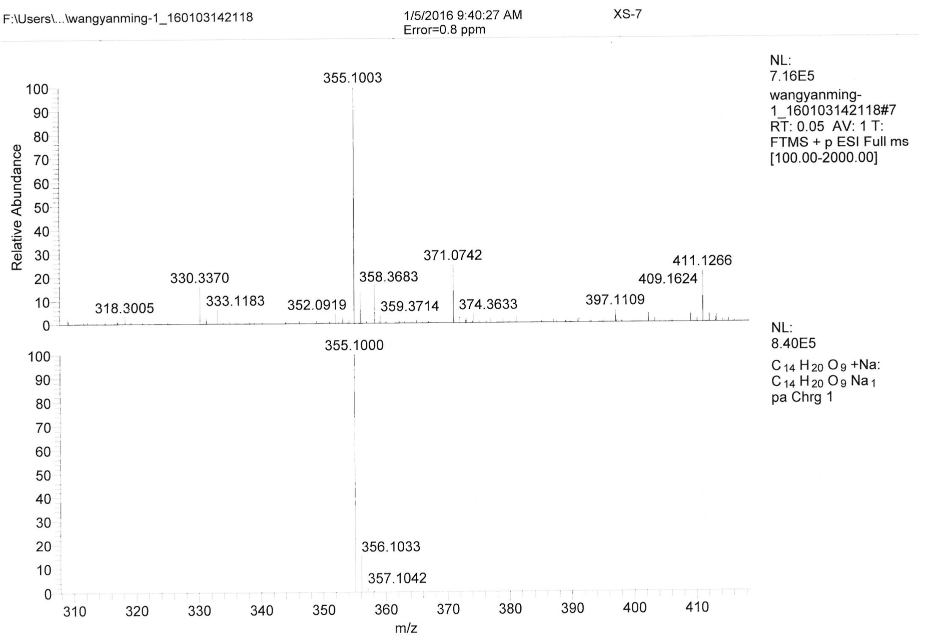

Figure S3. HR-ESI-MS of xspolyphenol A.

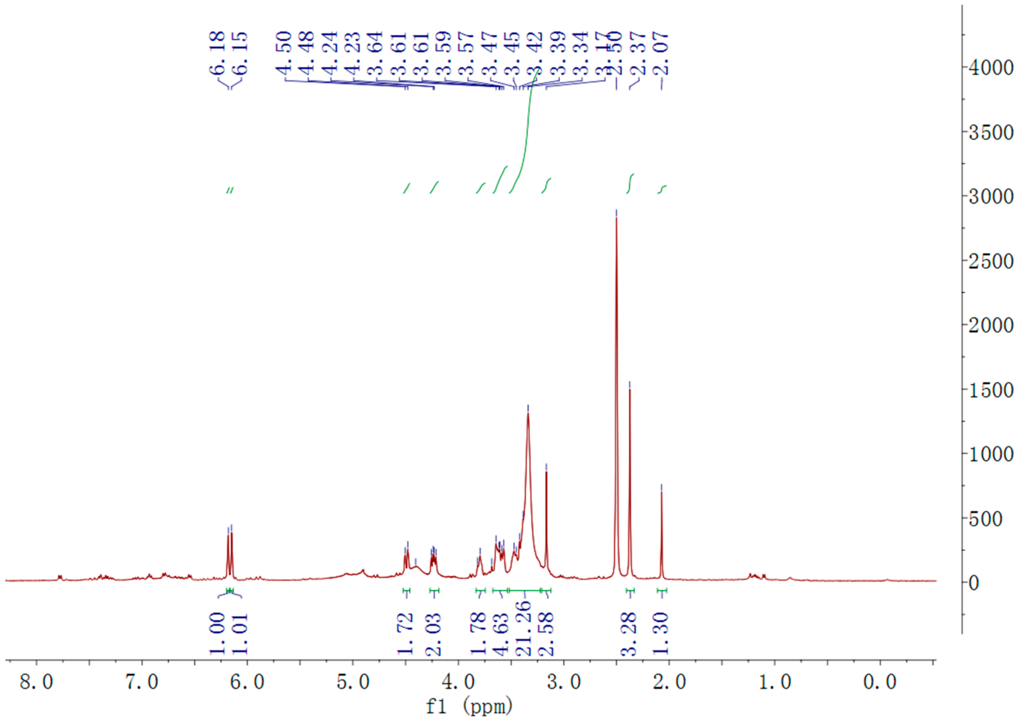

Figure S4. ^{1}H-NMR of xspolyphenol A.

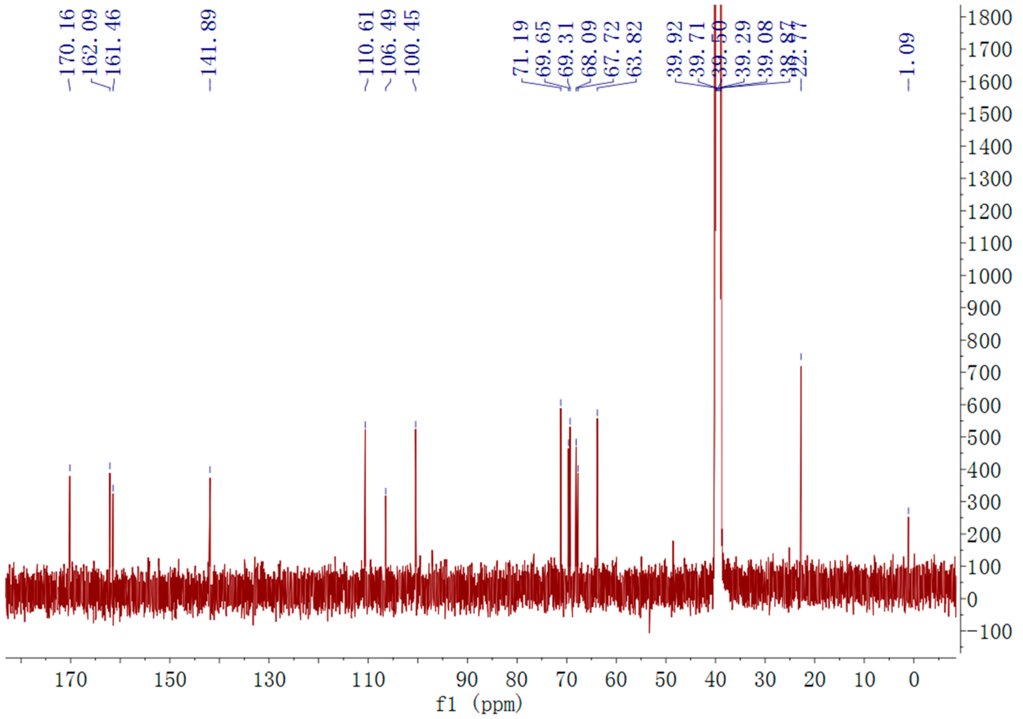

Figure S5. ^{13}C-NMR of xspolyphenol A.

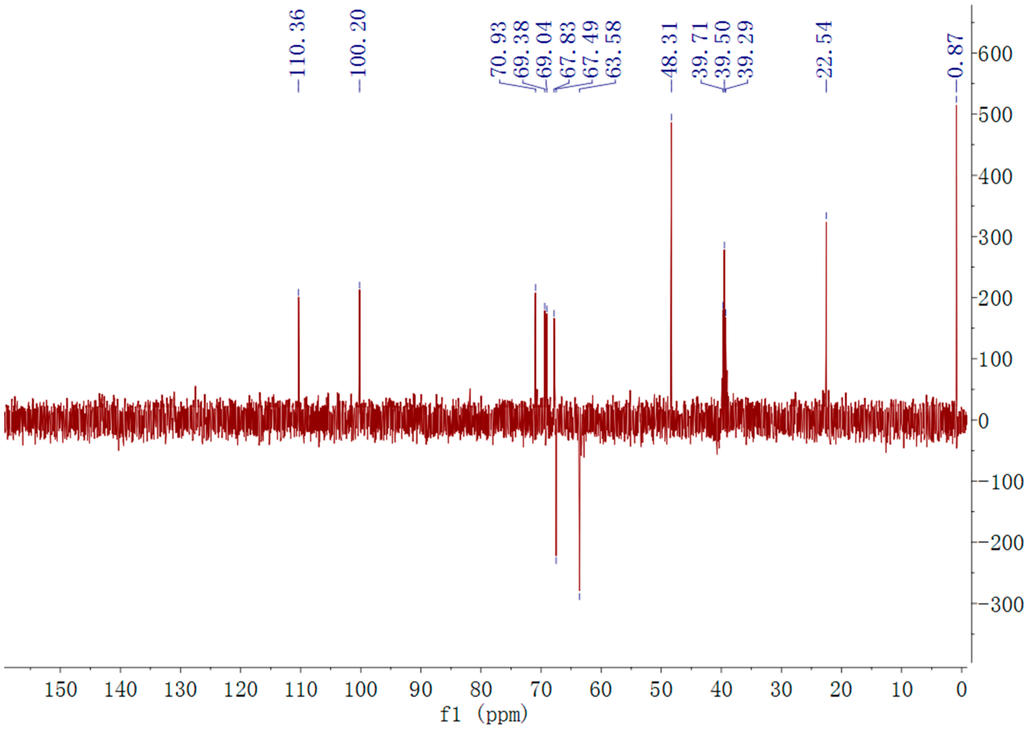

Figure S6. DEPT of xspolyphenol A.

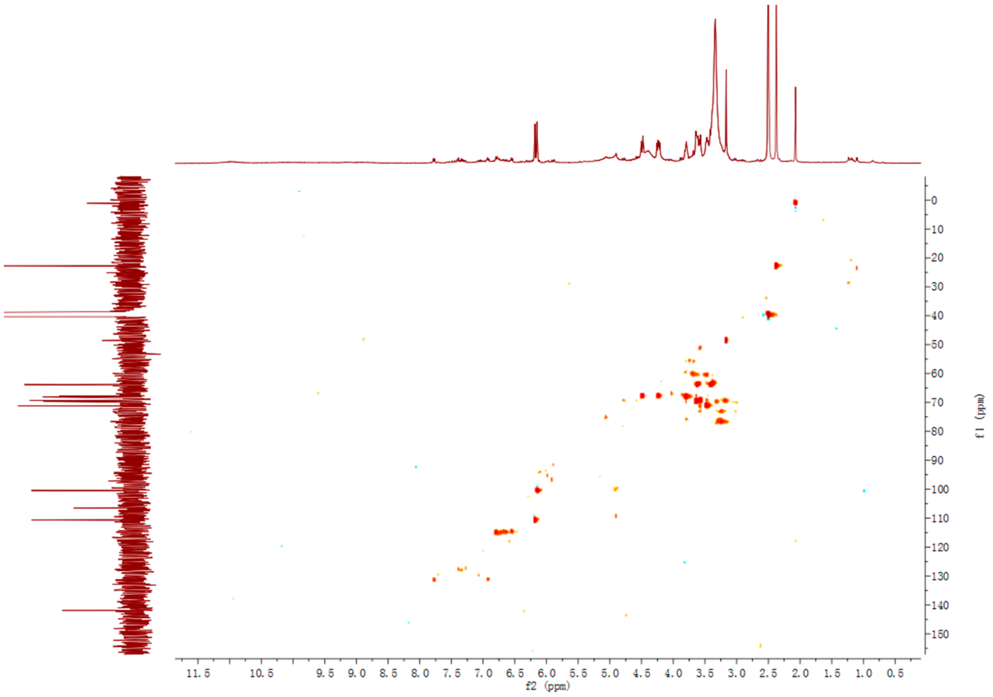

Figure S7. HSQC of xspolyphenol A.

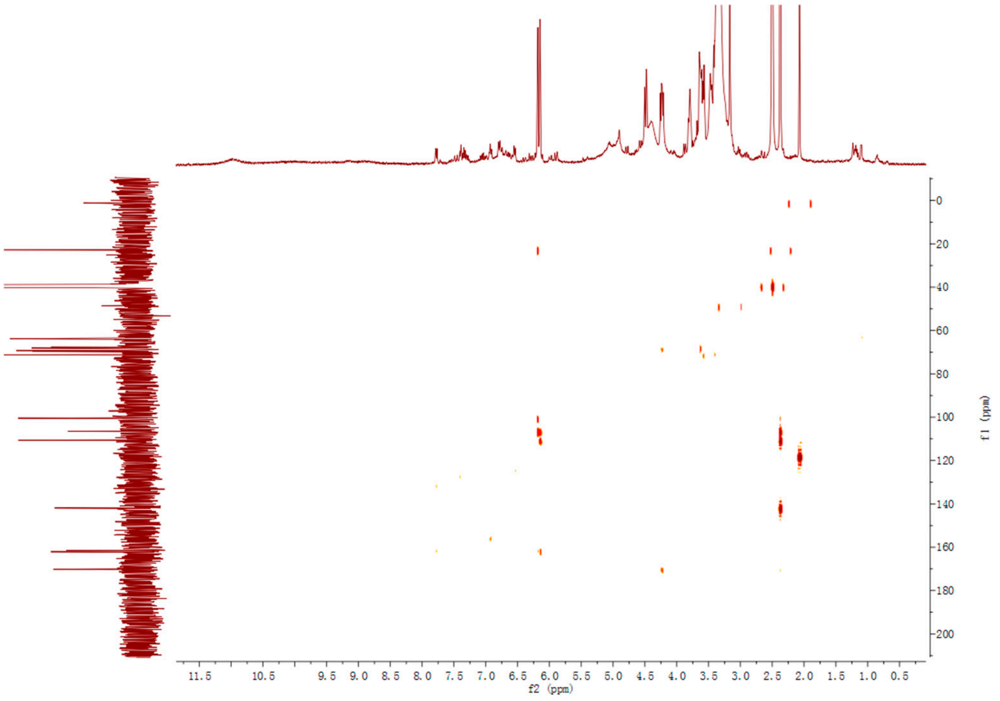

Figure S8. HMBC of xspolyphenol A.

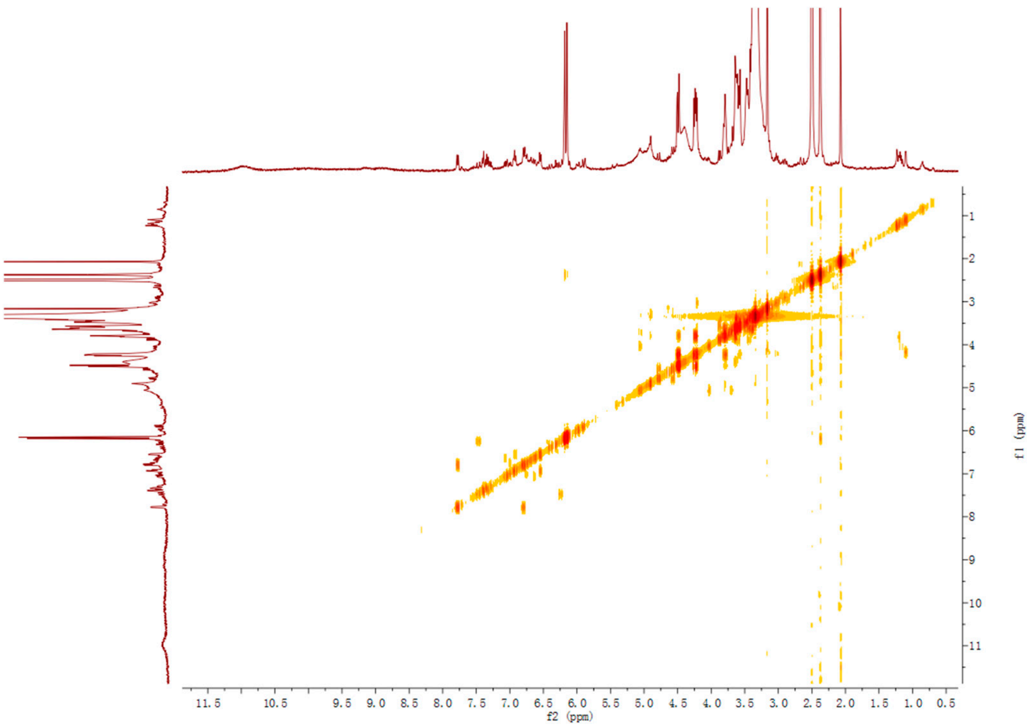

Figure S9. ^{1}H-^{1}H COSY of xspolyphenol A.

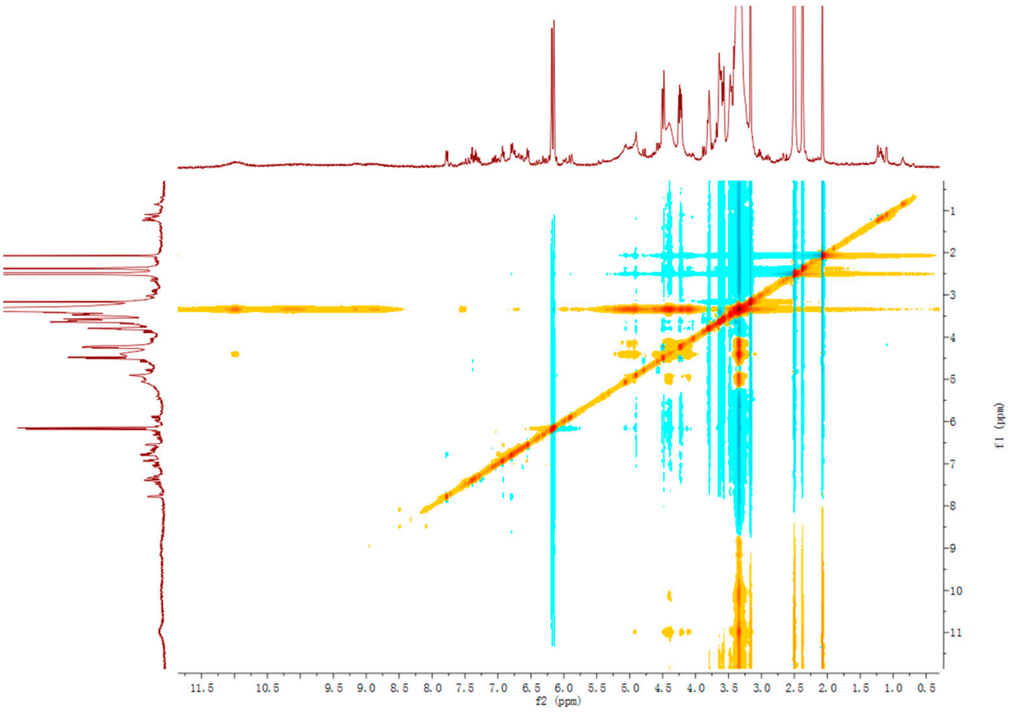

Figure S10. NOESY of xspolyphenol A.

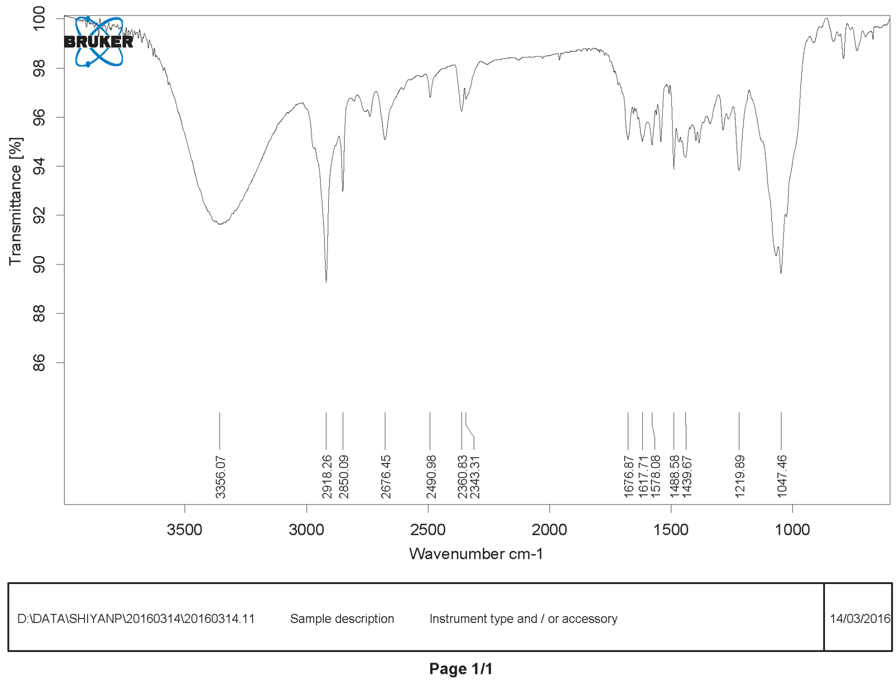

Figure S11. IR spectrum of xspolyphenol B.

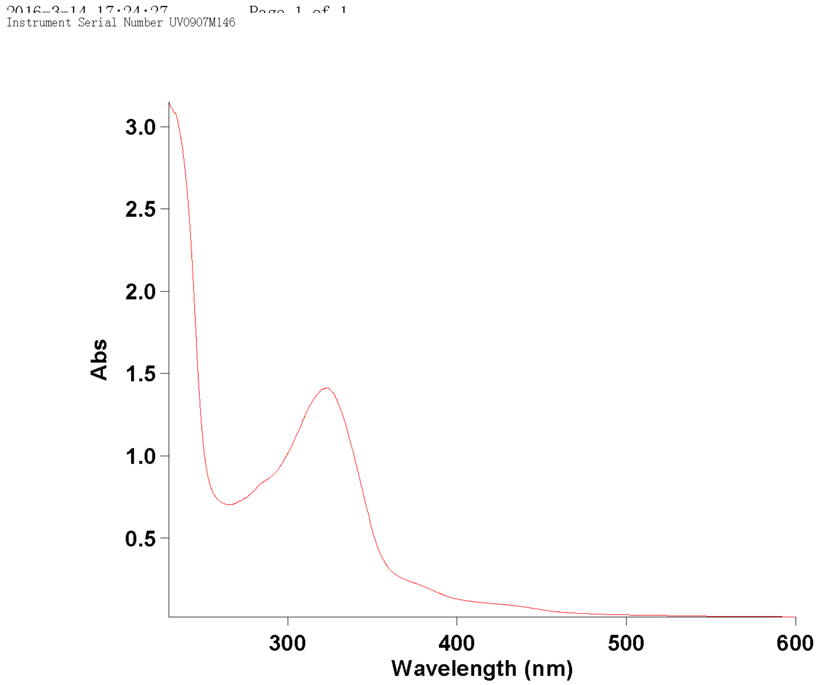

Figure S12. UV spectrum of xspolyphenol B.

Mass Spectrum SmartFormula Report

Analysis Info

Analysis Name	D:\Data\user\yangchunyan 20151214-1.d	Acquisition Date	12/14/2015 4:08:49 PM
Method	tune_low.m	Operator	BDAL@CN
Sample Name	M.W	Instrument / Ser#	micrOTOF-Q II 10453
Comment			

Acquisition Parameter

Source Type	ESI	Ion Polarity	Positive	Set Nebulizer	0.4 Bar
Focus	Active	Set Capillary	4500 V	Set Dry Heater	180 °C
Scan Begin	100 m/z	Set End Plate Offset	-500 V	Set Dry Gas	3.0 l/min
Scan End	1000 m/z	Set Collision Cell RF	300.0 Vpp	Set Divert Valve	Waste

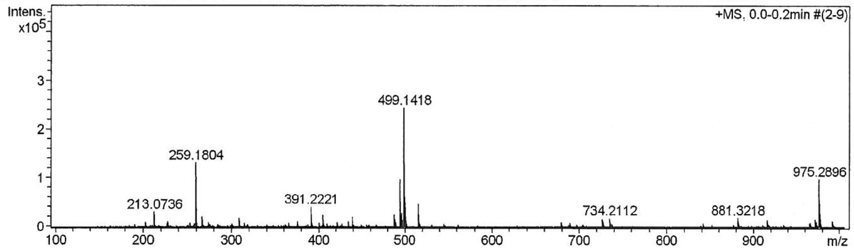

Meas. m/z	#	Formula	m/z	err [ppm]	Mean err [ppm]	rdb	N-Rule	e⁻ Conf	mSigma	Std I	Std Mean m/z	Std I VarNorm	Std m/z Diff	Std Comb Dev
499.1418	1	C 18 H 16 N 14 Na O 3	499.1422	0.9	1.1	17.5	ok	even	12.0	20.8	0.8	13.2	0.0	872.1
	2	C 20 H 28 Na O 13	499.1422	0.9	1.0	6.5	ok	even	14.5	26.7	0.5	9.0	0.4	786.6
	3	C 18 H 23 N 6 O 11	499.1419	0.3	0.4	10.5	ok	even	15.1	29.0	0.3	9.7	0.4	709.3
	4	C 16 H 11 N 20 O	499.1419	0.3	0.5	21.5	ok	even	16.2	27.9	0.7	18.2	0.1	881.6
	5	C 31 H 15 N 8	499.1414	-0.7	-0.4	28.5	ok	even	58.1	93.0	0.6	29.0	1.9	922.5
	6	C 33 H 20 N 2 Na O 2	499.1417	-0.1	0.2	24.5	ok	even	58.1	93.8	0.6	29.4	1.9	922.1

Bruker Compass DataAnalysis 4.0 printed: 12/16/2015 5:27:50 PM Page 1 of 1

Figure S13. HR-ESI-MS of xspolyphenol B.

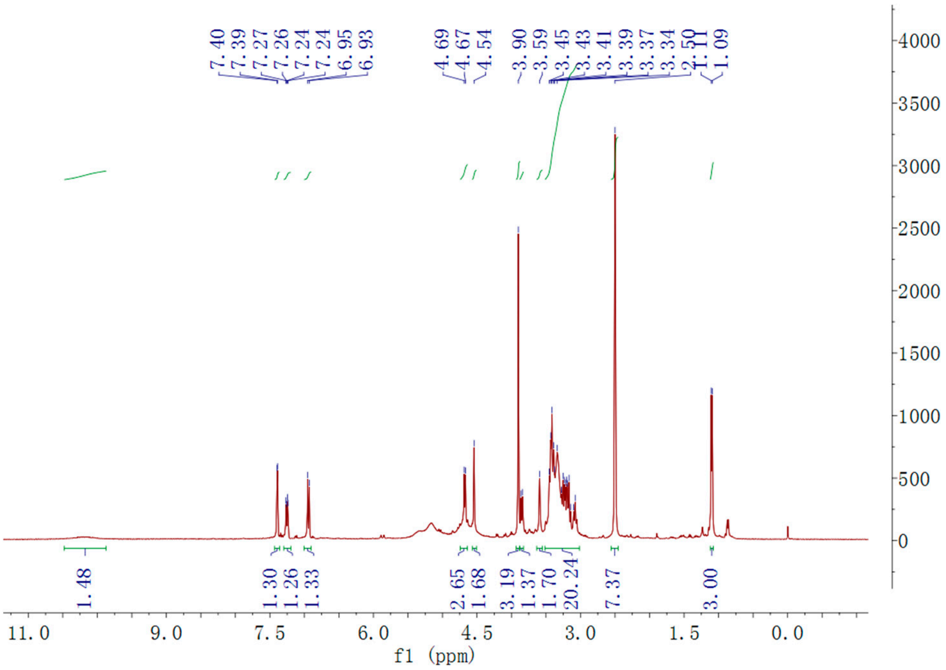

Figure S14. ^{1}H-NMR of xspolyphenol B.

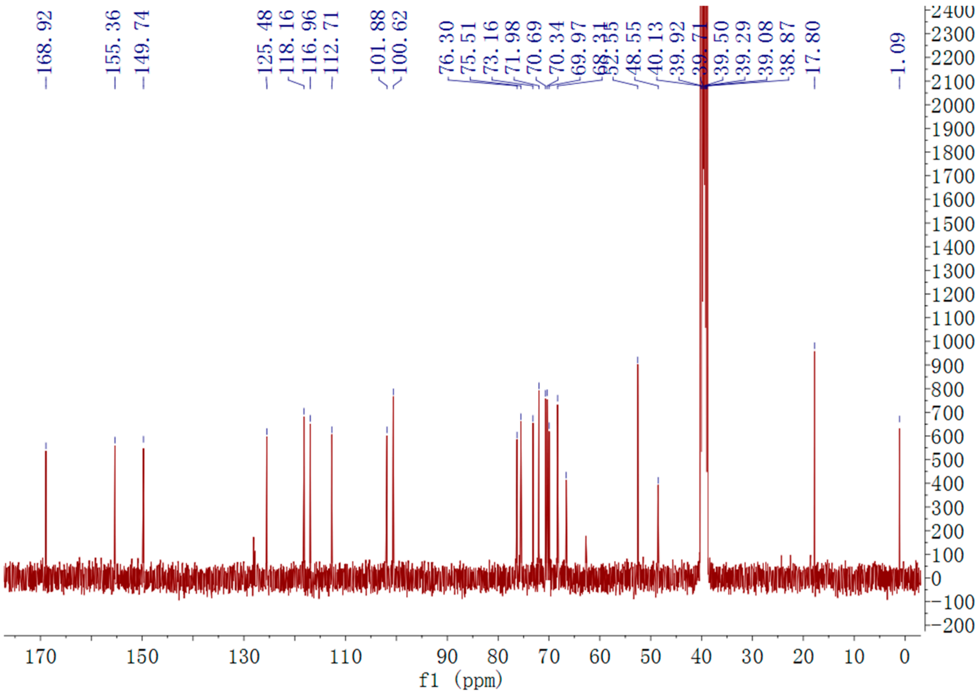

Figure S15. ^{13}C-NMR of xspolyphenol B.

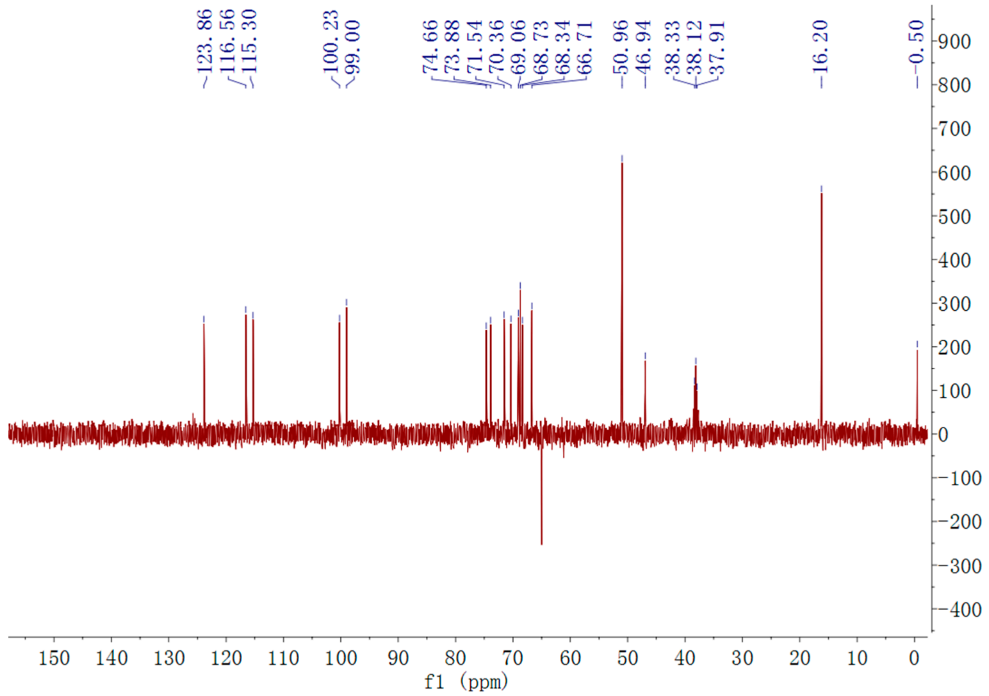

Figure S16. DEPT of xspolyphenol B.

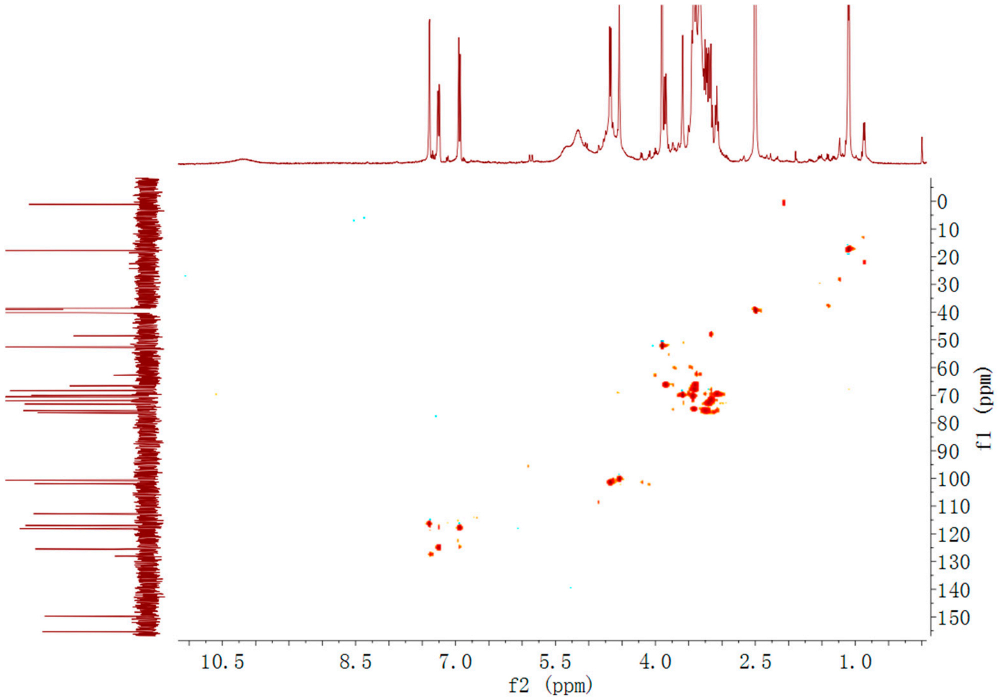

Figure S17. HSQC of xspolyphenol B.

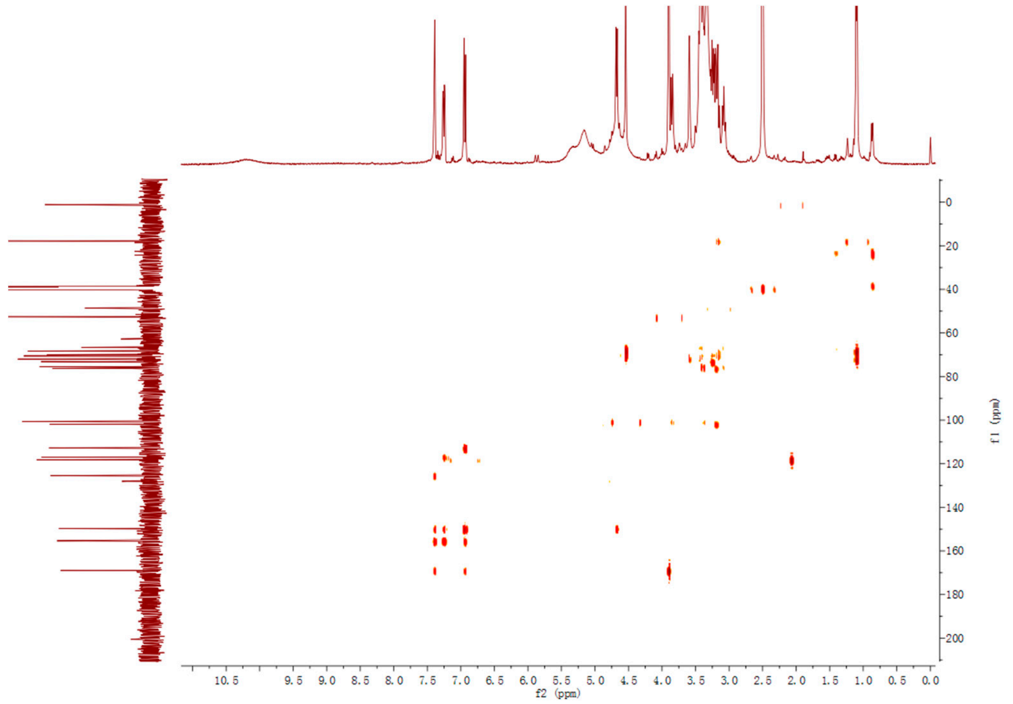

Figure S18. HMBC of xspolyphenol B.

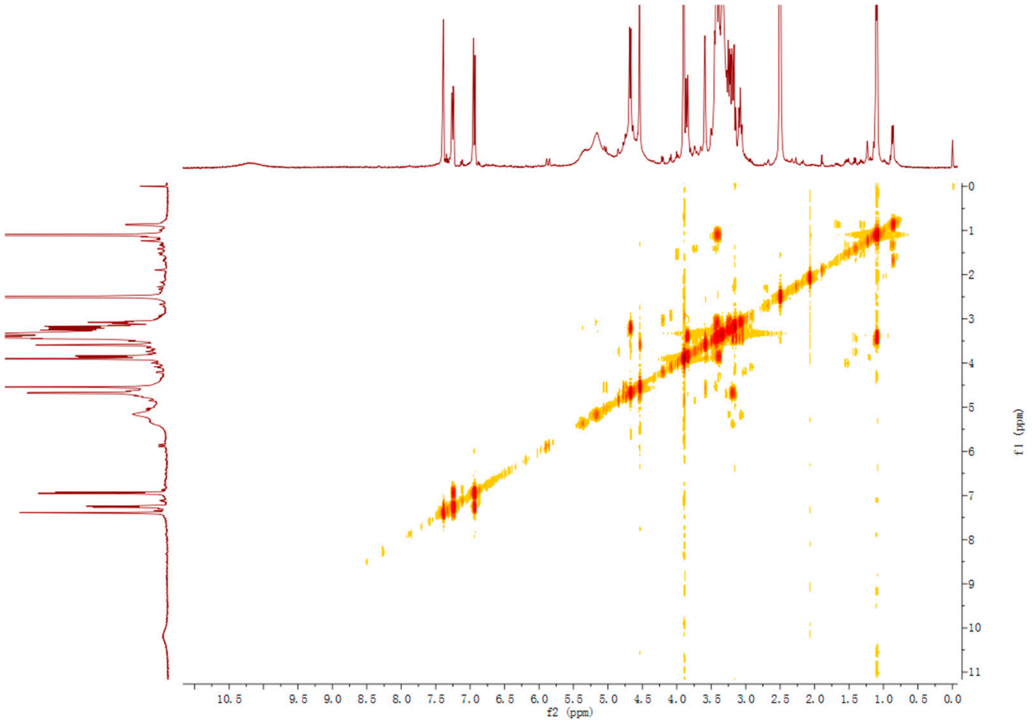

Figure S19. ^1H-^1H COSY of xspolyphenol B.

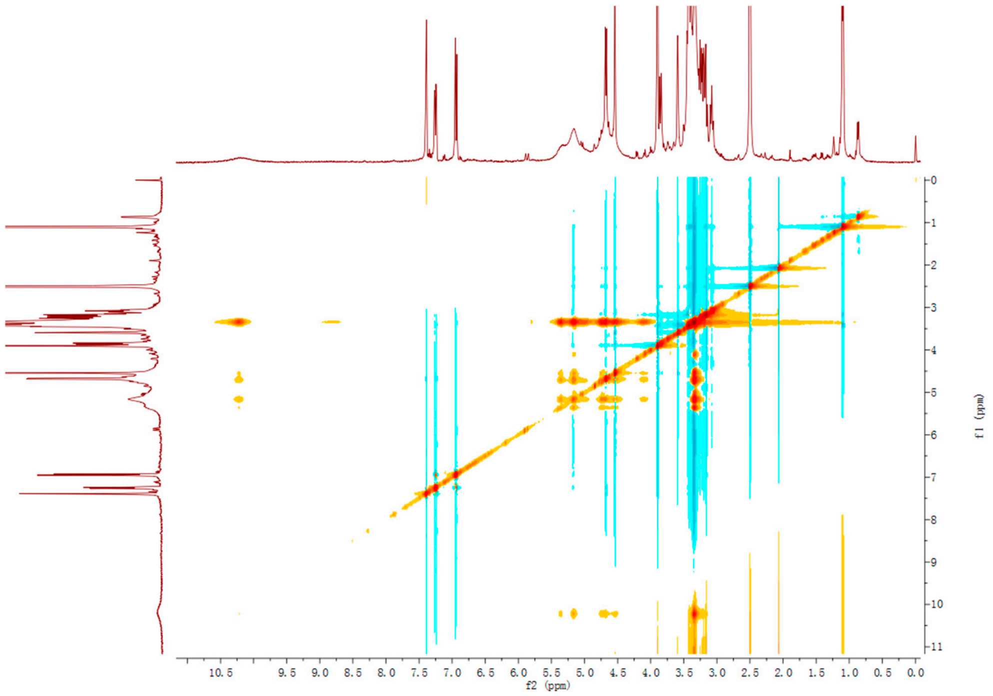

Figure S20. NOESY of xspolyphenol B.

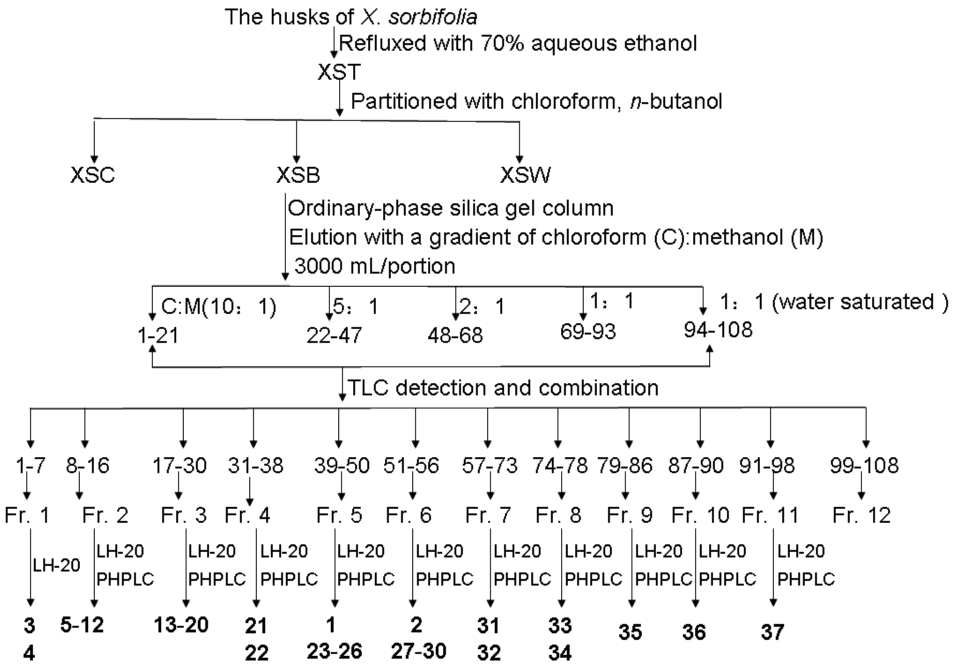

Figure S21. The extraction and separation diagram of polyphenols (**1**–**37**) from the husks of XS.
